# GAN-based underwater image enhancement and scene classification using transfer learning

**DOI:** 10.1371/journal.pone.0345593

**Published:** 2026-03-27

**Authors:** Amani Homoud, Saptarshi Das

**Affiliations:** 1 Mathematics Department, College of Science, Jazan University, Jazan, Saudi Arabia; 2 Centre for Environmental Mathematics, Faculty of Environment, Science and Economy, University of Exeter, Penryn Campus, Cornwall, United Kingdom; 3 Institute for Data Science and Artificial Intelligence, University of Exeter, North Park Road, Exeter, Devon, United Kingdom; Amrita Vishwa Vidyapeetham, INDIA

## Abstract

This paper provides an exploratory analysis of underwater video analysis techniques to enhance image quality and facilitate accurate classification of different marine species. Our methodology progresses through several steps, beginning with the quality of underwater images that might be reduced by variables such as decreased light intensity, color modification, and limited visibility. These attributes pose significant challenges to develop accurate object detection methods. This paper outlines the processing pipeline employed to enhance the quality of images from underwater videos and facilitate precise object detection. First, we use the Gray World (GW) algorithm for image enhancement, effectively mitigating the challenges of aquatic environment, such as color distortion and low contrast. Subsequently, we compare the traditional Histogram Equalization (HE) and the Contrast Limited Adaptive Histogram Equalization (CLAHE) algorithms to assess their efficacy in enhancing underwater image quality. Next, Canny Edge Detection is utilized to identify the prominent features in the enhanced images, aiding in subsequent classification tasks. Next, three state-of-the-art deep learning models, Visual Geometry Group 16-layer network (VGG16), 50-layer Residual Network (ResNet50), and 121-layer Densely Connected Convolutional Network (DenseNet121), are leveraged through transfer learning to classify underwater species, including fish, coral reefs, and sea turtles. Finally, by enhancing the visual quality of underwater images, our research contributes to better understanding of the underwater ecosystem and supports conservation efforts. Enhanced Super-Resolution GAN (ESRGAN) is a superior Generative Adversarial Network (GAN) technique to improve the quality of noisy images. This paper contributes to advancing the field of underwater image and video analysis, offering valuable insights for applications in marine biology, environmental monitoring, underwater robotics, and autonomous navigation.

## 1. Introduction

The ocean is a crucial component of the Earth’s surface that provides humanity with food, medicine, raw materials, tourism, and ecological research opportunities. Marine organisms play a crucial role in the oxygen cycle of the earth, climate regulation, and garbage decomposition, monitoring of fish biodiversity which are essential elements in marine research. Basic information for marine analysis includes fish abundance, species distribution, sex ratio, and behavioral traits. Scientists can also predict climate change in a particular area by analyzing changes in the population size of a specific marine species [1]. Furthermore, classifying fish helps monitor their movement patterns and migration trends, contributing to a more thorough understanding of different species. However, using oceanographers to manually process and analyze video data is time-consuming, subjective, and labor-intensive [2]. Therefore, this research on automated underwater fish detection and video analysis has promising future prospects [3]. Research based on extensive underwater data emphasizes the importance of using large robust, accurately labeled, and unbiased dataset [4]. The underwater environment typically presents several image processing challenges, such as low resolution, inadequate lighting, intricate backgrounds, obstruction, distortion due to camera movement, and the resemblance between various fish species [5,6].

Advanced algorithms improved the effectiveness of image processing models for underwater scene classification tasks as they prevent the model from becoming complex even for low-quality images [7]. Various enhancement techniques have been proposed in the scientific literature such as HE and the CLAHE algorithms, which aim to improve the overall contrast by redistributing pixel intensities. GW algorithm used for color correction attempts to compensate for wavelength dependent attenuation by averaging RGB (Red-Green-Blue) channels [8,9]. These methods are known for their computational efficiency and ease of implementation. However, they oprate at pixel level and often amplify noise under low-illumination conditions which are typically encountered in the underwater environment [10]. Recently, image enhancement techniques, particularly GAN has got increasing attention for their ability for enhancing images. GAN-based methods, such as SRGAN and ESRGAN have achieved massive improvement in the quality of blurry and noisy images [11], [12]. However, several works indicate that GAN-based enhancement may introduce some challenging color distortion and low contrast, as a side effect [13].

Deep learning approaches, particularly convolutional neural networks (CNNs) and their variants, are now the state of the art trends in fish identification research for automated image categorization [3]. CNNs were utilized in underwater video analysis on industrial scale farms and natural ocean environments [14]. Deep learning algorithms simplify accurate recognition, classification, and tracking of species by automatically learning complex patterns in large visual data. These models help fish farms automate routine tasks and identify abnormal behavior, increasing efficiency and reducing manual labor [15]. This paper helps in improving the quality of underwater images and simplify the accurate classification of marine species.

## 2. Previous works on underwater video processing

### 2.1. Model-based enhancement methods

Enhancing image, segmentation, tracking, and object detection are well established concepts. They are utilized in autonomous vehicles, smartphones, and surveillance purposes. Studies for enhanced underwater images provide an in-depth review of the methods used to address the challenges of capturing images in underwater environments. There are many methods, designed to deal with image enhancement, such as dark channel prior-based methods [16,17], polarization-based imaging [18,19], which aim to simulate and correct light propagation phenomena, such as water attenuation and scattering to fix color distortion and improve visibility. Moreover, histogram-based methods [20,21], retinex-based methods [22], fusion-based methods [23], and machine learning approaches [24], directly modify the captured image data to improve contrast, clarity, and color accuracy.

The Dark Channel Prior (DCP) approach is an enhancement technique that is employed to increase the quality of underwater images. The operation is based on the concept that in natural images, the dark channel typically consists of low-intensity values, indicating places not impacted by haze or underwater scattering [25]. DCP uses this characteristic to estimate the image without haze by examining the dark channel of the input image and removing the haze component. This method improves underwater images’ clarity and sharpness by efficiently minimizing haze and turbidity impact. The DCP method is widely used in underwater image processing because it effectively enhances image quality under difficult underwater conditions [26]. The study in [27] showcased the efficacy of the DCP approach in improving the quality of underwater images and emphasized its significance in underwater imaging. While, the authors in [28] demonstrated that it is impossible to eliminate the haze, and as a result, images produced by these approaches often exhibit a color cast, typically green or blue to absorb and scatter light in an underwater environment. Polarization-based imaging, as described in [18], utilizes simulations of light interactions with water molecules to correct color changes and improve visibility in underwater videos of fish and turtles. Polarization-based imaging methods utilize the polarization characteristics of light to improve underwater visibility and minimize glare. By analysing the polarization patterns of light reflected from underwater objects, these techniques can eliminate unwanted reflections and improve the distinction between different elements in underwater video footage [29]. Polarization-based imaging provides an efficient method to enhance visibility and improve the sharpness of underwater images, which makes it extremely valuable for recording intricate footage of marine species.

Retinex-based approaches are influenced by the human visual system’s capacity to perceive color constancy under different lighting conditions. The techniques described in [30] involve breaking down an image into its reflectance and illumination elements. By increasing the reflectance, color distortions can be corrected and the contrast can be improved. Fusion-based methodologies combine data from various images or distinct processing procedures to get a single improved image. This method can involve combining images taken under different lighting conditions or integrating results from several enhancement algorithms to produce enhanced visibility and accurate color representation in underwater images [23]. Together, these intangible enhancement techniques offer efficient options for improving the sharpness and complexity of underwater images, enabling more accurate observation and analysis of marine organisms, such as fish and turtles. A comparison of the literature survey is summarized in Table 1.

**Table 1:** Comparison of underwater image enhancement methods.

Reference	Method	Main Research Focus
**[31,30]**	Retinex-based methods	Image enhancement and improving low-quality images
**[32]**	Retinex theory	Improves the contrast, sharpness, color constancy, and dynamic range of degraded underwater visuals
**[8,23,33]**	Fusion-based algorithm	Enhance underwater images across various cameras, depths, and lighting situations
**[16]**	Generalization of the Dark Channel Prior Methods (GDCP)	Reduced clarity, modified color balance, and reduced contrast
**[10]**	Dehazing methods	Enhance the quality of image and video and provide high image contrast
**[34,24]**	Generative Adversarial Network (GAN)	Enhance underwater images by focusing on perceptual quality improvements, considering aspects such as color, texture, and style
**[35,36]**	CycleGAN	Color correction
**Current work**	Gray World	Improving color imbalance
**Current work**	Histogram Equalization	Enhancing the visual of underwater images
**Current work**	Contrast Limited Adaptive Histogram Equalization	Improve contrast and image quality
**Current work**	Enhanced Super-Resolution GAN (ESRGAN)	Improve the visual image quality

### 2.2. Contrast and color correction enhancement methods

Underwater video analysis is a rapidly developing field with diverse applications that range from marine biology research to underwater robotics and environmental monitoring. Existing methods and techniques in this domain encompass a wide range of approaches aimed at enhancing image quality, detecting objects, and classifying marine organisms. Traditional techniques often rely on handcrafted features and image processing algorithms, while more recent advances leverage on deep learning algorithms for automated analysis.

One common challenge in underwater video analysis is poor visibility caused by factors such as water turbidity, light attenuation, and color distortion. To address this, researchers have proposed various image enhancement techniques tailored to the underwater environment. These include methods based on HE [21], CLAHE [37], and GW algorithm [9]. HE and CLAHE modify the intensity distribution of an image to enhance contrast and visibility [21]. The techniques mentioned above improve overall visual quality by distributing the most common intensity values, enhancing the visibility of details in underwater scenes [38]. While these techniques can improve image quality to some extent, they often struggle with preserving color fidelity and enhancing fine details, especially in highly turbid or low-light conditions [39].

### 2.3. Deep learning for video enhancement and classification

Deep learning methods are frequently applied to many scenarios in computer vision and video data analysis. Recent works have explored GAN-based enhancement methods, such as SRGAN and ESRGAN, which are intended to improve fine details and enhance visual image quality [40]. While these methods produce visually appealing results, several studies indicate that optimization using competitive networks can introduce artificial textures and fail to accurately improve recognition in underwater environments, mainly due to discrepancies with real training data [40]. Deep learning has become essential method for classifying underwater organisms due to limited data available for classification. CNN architectures, such as ResNet, DenseNet, and VGG have been widely studied [41], while DenseNet model showed improved features [7]. These models have adopted their ability to take advantage of pre-training features from large datasets [42], and achieved improvements in classification accuracy between underwater datasets [7]. Recent studies include temporal modeling of streaming video data for understanding the overall context of underwater scenes. However, these models introduce increasing computational complexity and are critical to deploy in resource limited underwater environment.

## 3. Motivation and novelty

Our current research is pursued through a novel strategy that involves a series of sequential computational steps. Firstly, we conducted video exploration to collect a variety of underwater footage from various marine environments and conditions. Secondly, we employ advanced image enhancement algorithms, specifically the GW algorithm, to improve the visual quality of underwater images. This step focuses on resolving typical problems such as color distortion and low contrast to improve the clarity of the footage. Following image enhancement, a comparative analysis is performed on two commonly used techniques: HE and the CLAHE algorithm [37], to evaluate their effectiveness in improving image quality. Additionally, the Canny edge detector is used to find important features in the enhanced images, which helps with the classification tasks. Next, we utilize deep learning models with transfer learning in well-known architectures, including VGG16, ResNet50, and DenseNet121 to classify marine organisms, including fish, coral reefs, and sea turtles. Finally, we utilize the ESRGAN technique to improve the visual quality of underwater images.

In summary, this paper makes the following contributions:

- We enhance the quality of underwater images of fish species.
- We improve the classification of underwater species, especially fish, sharks, and coral reefs using the latest techniques in ecological image/video analysis.
- We test different algorithms to distinguish and show structural differences between different species.

## 4. Proposed methodology

**Fig 1:**
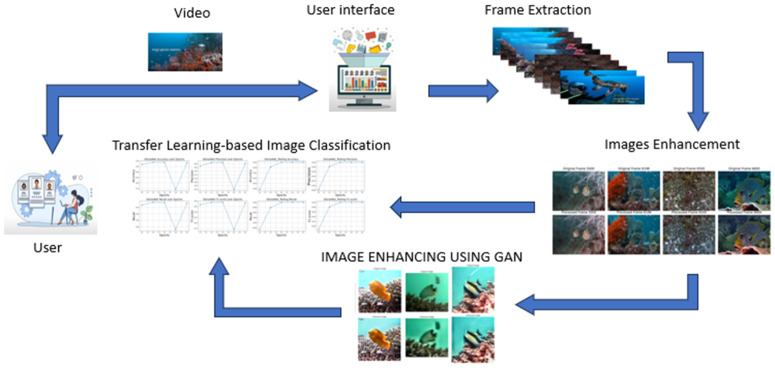
Schematic diagram of the proposed underwater video analysis pipeline. It starts with input video and preprocessing, enhancement techniques to improve image quality and passed to classification model, which outputs the final label.

Fig 1 shows a flow chart that outlines the steps of the computational experiments. Initially, all videos were decomposed into individual images. To prevent the absence of any fish-related data, we extracted all individual frames, each with its appropriate annotation. Both frames and annotations are stored in different folders. The second phase involves converting the initial annotation into the appropriate format for CNN designs such as VGG16, ResNet50, and DenseNet121 models, followed by applying data cleaning to the entire dataset.

### 4.1 Data-set description

**Fig 2:**
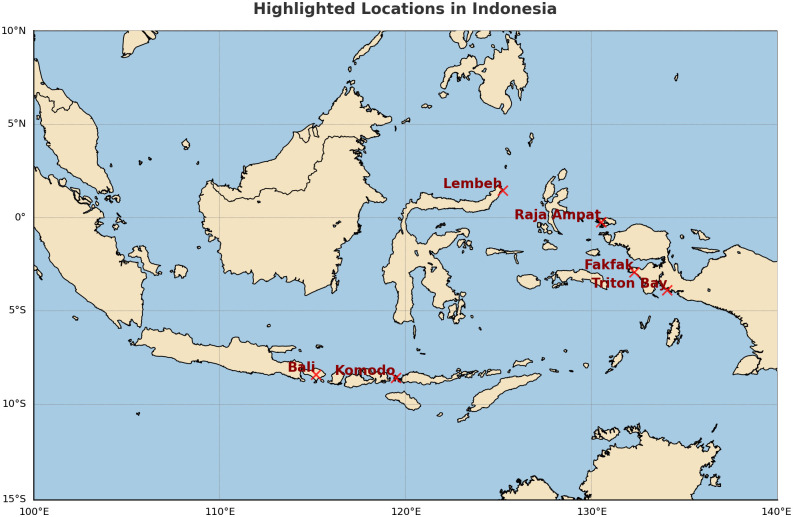
Geographical locations for the Indonesia’s islands Bali, Komodo, Raja Ampat, Lembeh, Fakfak, and Triton Bay. This spatial distribution maps were generated using a Python-based geospatial pipeline with Matplotlib to visualize the Indonesia region.

**Fig 3:**
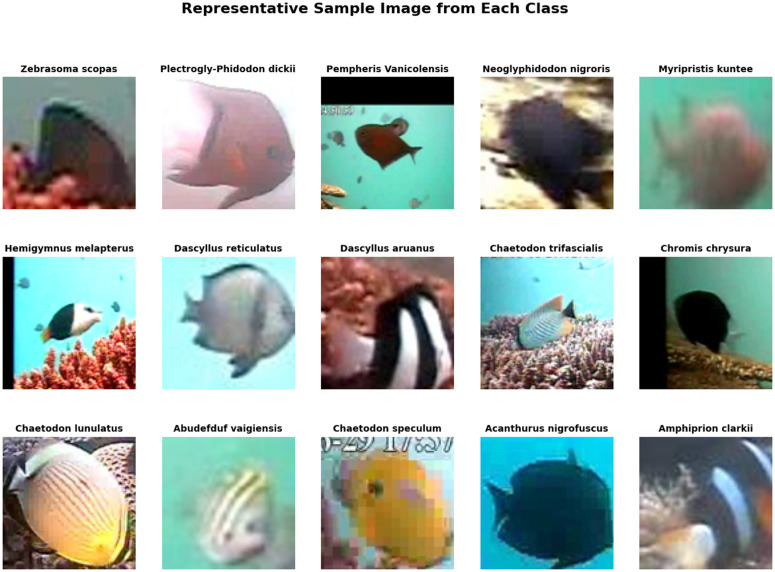
Sample image from each class in the LifeCLEF-2015 dataset. It contains 15 classes of underwater species [44].

In this study, we used the underwater video dataset that sourced from Undersea Production website [43], which is a professional underwater film production company. The video was recorded in Indonesia and covered locations such as Bali, Komodo, Raja Ampat, Lembeh, Fakfak, and Triton Bay as shown in Fig 2. These regions contain rich marine species that help us study fish diversity and classify fish species. The dataset contains a high-resolution video featuring various species in different environment conditions such as lighting conditions, turbidity, underwater species types, etc. We also used the LifeCLEF-2015 dataset [44] for enhancement techniques. This dataset is challenging because it has low image quality and class similarity as shown in Fig 3. We consider here in this study three different types of underwater species such as fish, coral reefs, and turtles that the data comes from the video (fish, coral reef, and turtle) and the LifeCLEF-2015 include many types of fish. The dataset includes 9,162 annotations across 15 species, but the distribution is strongly imbalanced because *Dascyllus reticulatus* has 3,165 species, *Chaetodon lunulatus* with 1,217 and *Pempheris vanicolensis* with 999. Some species, such as *Neoglyphidodon nigroris* with 85 and *Zebrasoma scopas* with 72 as shown in Table 2, are severely underrepresented, making them challenging for deep learning models to learn. This imbalance has direct implications for detection and classification performance, particularly for rare or visually ambiguous species.

**Table 2:** Species Annotation Distribution in the LifeCLEF-2015 Dataset.

Species	Annotations
**Dascyllus Reticulatus**	3165
**Chaetodon Lunulatus**	1217
**Pempheris Vanicolensis**	999
**Dascyllus Aruanus**	894
**Plectrogly-Phidodon Dickii**	737
**Amphiprion Clarkii**	363
**Chaetodon Trifascialis**	335
**Acanthurus Nigrofuscus**	294
**Chromis Chrysura**	275
**Hemigymnus Melapterus**	242
**Myripristis Kuntee**	214
**Chaetodon Speculum**	138
**Abudefduf Vaigiensis**	132
**Neoglyphidodon Nigroris**	85
**Zebrasoma Scopas**	72
**Total**	**9162**

### 4.2. Video data exploration

**Fig 4:**
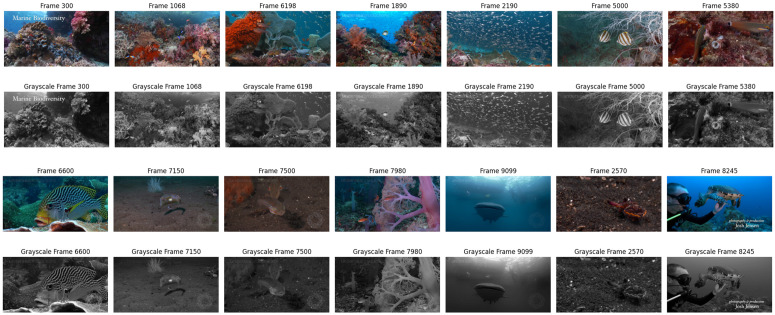
Several illustrations frames. The frames selected from the underwater video, row 1 and 3 display original images, row 2 and 4 display images are in grayscale. This shows how grayscale converting removes color and retains intensity and important details in the image.

**Fig 5:**
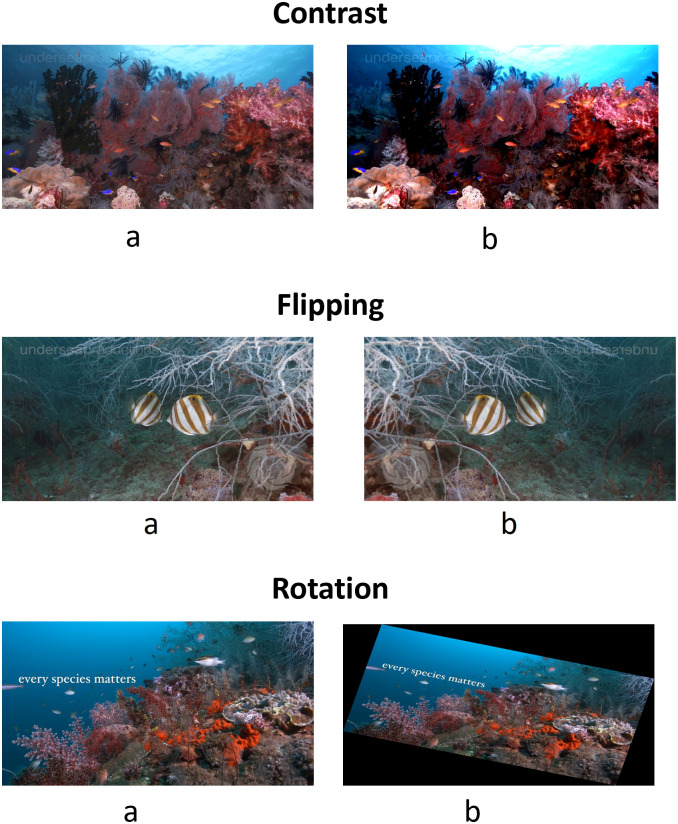
Data augmentation techniques. First row: a) original image, b) contrast, second row: a) original image, b) flipping, and third row: a) original image, b) rotation. This helps to improve model learning and performance.

In the initial step of our methodology, we conducted video exploration to gain insights into the characteristics of the underwater footage. This entailed examining the metadata linked to the video, such as frame count, frame size, and frame rate. The information indicated that the video consisted of 9940 frames, each with a width of 1920 pixels and a height of 1012 pixels. The footage was captured at a frame rate of 59.94 frames per second. We visually inspected the video by plotting a selection of the frames to create representative samples of the footage. 14 frames, selected from the video are displayed in Fig 4, providing a snapshot of the underwater world captured in the footage. This initial study laid the foundation for the subsequent data processing and analysis phases. These frameworks provide valuable context for understanding the challenges and opportunities for the analysis of underwater video and guide the subsequent steps described in the methodology, including image enhancement and classification tasks. In the last step before image enhancement, we use the augmentation techniques to improve the robustness and efficiency of the model when dealing with underwater images. Underwater conditions such as turbidity, low lighting conditions, and color distortions make it crucial to expose the model to a wide variety of training images [45]. Data augmentation techniques, such as rotation, flipping, and contrast adjustments help simulate different environmental conditions and make the model more adjustable as shown in Fig 5.

### 4.3. Image enhancement methods

**Fig 6:**
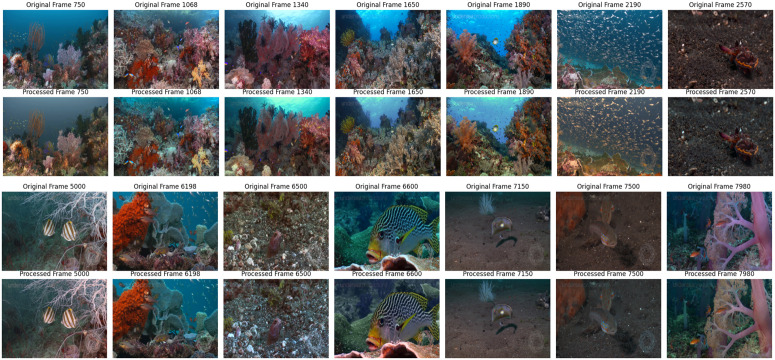
Underwater sample frames. The frames selected from underwater video with clear environments, row 1 and 3 show original images and row 2 and 4 show images obtained by the GW algorithm.

**Fig 7:**
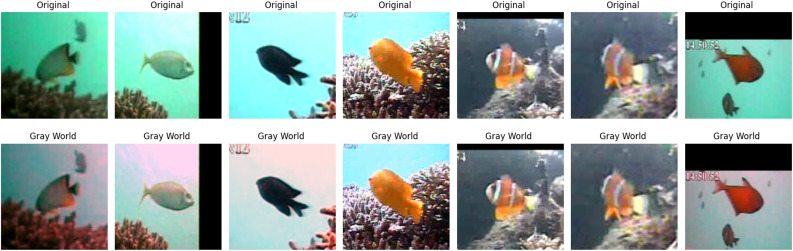
Example for complex underwater environments. The complex underwater environment such as low visibility due to turbidity, inconsistent lighting, and noisy background. Row 1 shows original images and row 2 shows images obtained by the GW algorithm, which correct the color imbalance. This improves color and visual quality of the images.

We utilize the GW algorithm to improve color distortion and contrast in underwater images. It assumes that in ideal illumination, an image should appear neutral gray with equal intensities for the red, green, and blue channels. First, the algorithm calculates the mean intensity of each color channel and the average intensity of the image. Each channel’s mean is scaled proportionally to match the overall average, neutralizing color casts. The adjusted values are clipped to stay within the valid range [0, 255] [39]. The GW algorithm is simple to use and effective with various color distributions. Its performance may be limited in images with a single color tone, since the assumption of a neutral gray may not hold. However, it is useful for improving color imbalances and preprocessing images for future analysis or augmentation. The visualization of the GW algorithm for underwater video is shown in Fig 6. For complex underwater environments, we used the LifeCLEF-2015 dataset [44] as shown in Fig 7. The equations for mean intensity of each channel is computed as follows [39]:


$$\begin{array}{c} \begin{array}{cc} \mu_{R} & =\frac{1}{N}\sum_{i,j}I_{R}(i,j),\;\mu_{G}=\frac{1}{N}\sum_{i,j}I_{G}(i,j),\\ \mu_{B} & =\frac{1}{N}\sum_{i,j}I_{B}(i,j), \end{array}\; \end{array}$$ (1)

where, *N* is the total number of pixels in the image, *I*_*R*_, *I*_*G*_, and *I*_*B*_ are the channel intensities.

The global gray reference value is then computed as:


$$\mu_{avg}=\frac{\mu_{R}+\mu_{G}+\mu_{B}}{3}.$$ (2)

The corrected image channels are obtained by:


$$\begin{array}{cc} \overset{\prime }{\underset{R}{I}}(i,j) & =I_{R}(i,j)\cdot \frac{\mu_{\text{avg}}}{\mu_{R}},\;\overset{\prime }{\underset{G}{I}}(i,j)=I_{G}(i,j)\cdot \frac{\mu_{\text{avg}}}{\mu_{G}},\\ \overset{\prime }{\underset{B}{I}}(i,j) & =I_{B}(i,j)\cdot \frac{\mu_{\text{avg}}}{\mu_{B}}, \end{array}$$ (3)

where, $\frac{\mu_{\text{avg}}}{\mu_{R}}$, $\frac{\mu_{\text{avg}}}{\mu_{G}}$, and $\frac{\mu_{\text{avg}}}{\mu_{B}}$ are the scaling factors for each channel while,


$$I_{\text{channel }}^{\prime }=\min\left(255,\max\left(0,I_{\text{channel }}^{\prime }\right)\right).$$ (4)

### 4.4. Histogram equalization vs. CLAHE

In this step, we conducted a comparative analysis between HE and CLAHE to evaluate their effectiveness in enhancing the visual quality of underwater images. HE is a widely used technique in image processing that aims to improve contrast by redistributing pixel intensities such that the histogram of the output image becomes approximately uniform. It operates globally on the intensity values of the image without considering local neighborhoods. CLAHE is an adaptive method that divides the image into small tiles and applies histogram equalization to each tile separately. This adaptive approach prevents over-amplification of noise in regions with low contrast, which can occur with standard histogram equalization. For our analysis, we applied HE and CLAHE to each underwater video frame. The HE process involved using *OpenCV*’s *‘equalizeHist’* function to equalize the intensity histogram of the RGB channels independability. On the other hand, for CLAHE, we utilized the *‘createCLAHE’* function to create a CLAHE object with specified parameters and applied it to the $L^{*}$ channel of the $L^{*}a^{*}b^{*}$ color space that represents colors using three channels: $L^{*}$ for lightness, and $a^{*}$ and $b^{*}$ channels for correct color cast (green/blue) [20]. The resulting frames from both methods were visually inspected and compared to assess their performance in enhancing the image contrast and visibility of underwater features. We present the results of our analysis through the following examples.

**Fig 8:**
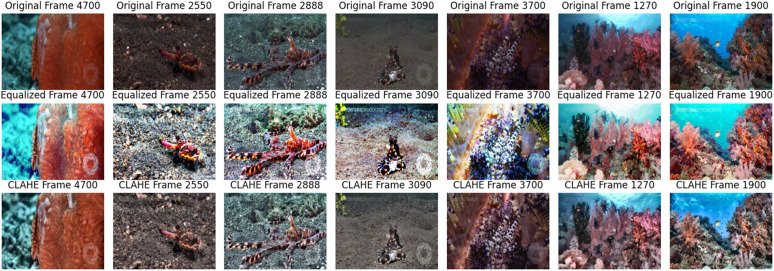
Examples images of HE and CLAHE. The images showing the resulting frames after applying HE, CLAHE and the corresponding original frames. This shows contrast enhancement in two techniques, HE improves overall contrast, while, CLAHE improve the local details.

Fig 8 provides visual representations of the enhancement achieved by each method, aiding in our comparative analysis. This comparative analysis provides insights into the strengths and limitations of each method and helps in determining the most suitable approach for enhancing underwater imagery.

### 4.5. Canny edge detection

**Fig 9:**
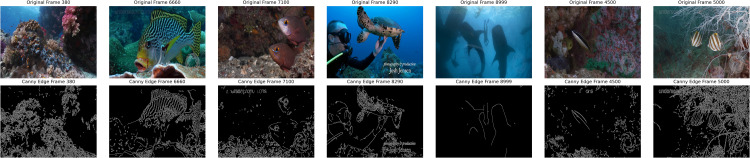
Canny edge detection frames. Row 1 shows the original frames. Row 2 shows the Canny edge detected frames, which highlight the fine edges with reducing noise to detect clear boundaries.

**Fig 10:**
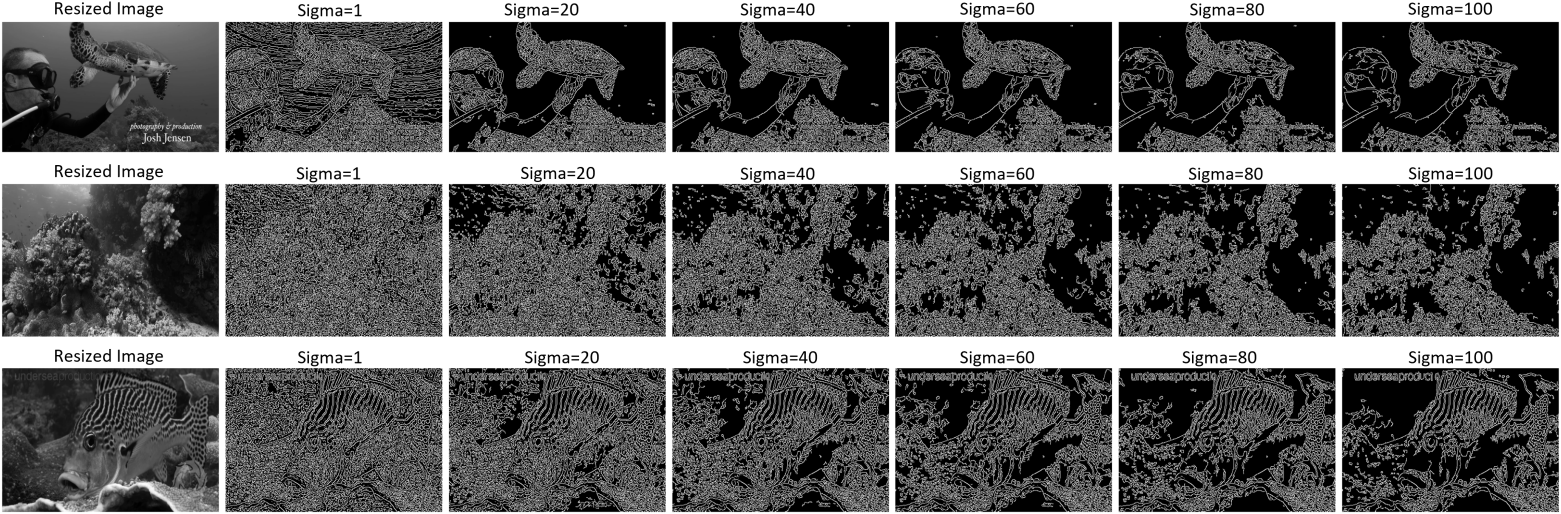
Samples of edge detector. Edge detector for turtle, coral reef, and fish images are shown with varying *σ*. Small value of *σ* preserves fine details, while higher *σ* reduces the noise and less details of the original structures. Comparisons are made on different noise levels with *σ* = 1, *σ* = 20. *σ* = 40, *σ* = 60, *σ* = 80, and *σ* = 100.

In this step, we employed the Canny edge detector to identify the edges in underwater images. Fig 9 presents the edge-detected frames alongside the original frames. This visual representation aids in analysing the effectiveness of the Canny edge detector in identifying underwater features and structures. Edge detection is a fundamental technique in image processing that aims to identify abrupt changes in brightness, which typically correspond to object boundaries or significant features within the image as shown in Fig 10. The Canny edge detector, introduced in [46], is renowned for its accuracy and robustness. It operates by first smoothing the image with a Gaussian filter to reduce noise. Subsequently, it computes the gradient magnitude and direction at each pixel using derivatives of Gaussian filters. Finally, it performs non-maximum suppression to thin out the detected edges and applies hysteresis thresholding to determine the final edge pixels. Mathematically, the process involves convolving the image with a Gaussian kernel to obtain the smoothed image [47]:


$$G(x,y)=\frac{1}{2\pi \sigma^{2}}e^{-(\frac{x^{2}+y^{2}}{2\sigma^{2}})}.$$ (5)

Equation (5) represents the Gaussian kernel and σ is the standard deviation of pixel intensities. Next, equation (6) represents the gradient magnitude and direction θ which are computed as:


$$|G|=\sqrt{(G_{x}{)}^{2}+(G_{y}{)}^{2}},$$ (6)

$$\theta =\text{arctan2}(G_{y},G_{x}).$$ (7)

**Fig 11:**
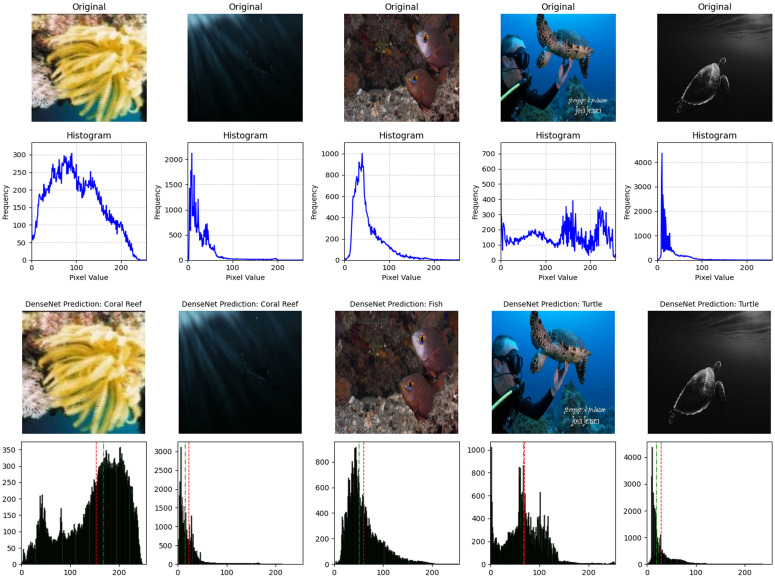
Histograms of images with different contrast levels. The images of different contrast such as dark image, bright image, color image, and high or low contrast images.

Here, *G*_*x*_ and *G*_*y*_ are the derivatives of the smoothed image in the horizontal and vertical directions, respectively. Finally, the hysteresis thresholding is applied to determine the final edge pixels, where a high threshold is used to identify strong edge pixels and a low threshold is employed to link weak edge pixels to the strong ones. By implementing the Canny edge detector, we could visualize the detected edges in the underwater images. This technique is invaluable for extracting essential features and structures from the imagery, facilitating subsequent analysis tasks such as object detection and classification. Here, Fig 11 shows the histogram of different contrast images and the pixel values to analyze the intensity levels.

## 5. Transfer learning models, training and performance evaluation

In this step, we employ transfer learning to fine-tune the pre-trained CNN models, namely VGG16, ResNet50, and DenseNet121, for the image classification task between fish, coral reef, and sea turtle. Each pre-trained model was initialized with pre-trained weights in the ImageNet dataset [48]. We customized the models by adding additional layers for classification. Specifically, we appended a flatten layer to the output of the pre-trained models, followed by one or more dense layers. These dense layers utilized Rectified Linear Unit (ReLU) activation functions to introduce nonlinearity and a final dense layer with softmax activation to output class probabilities. The final processed data set consisted of approximately 9,940 images divided into 80% training, 10% testing, and 10% validation.

The training data, consisting of 3,000 images per class, was utilized to fine-tune these models. We applied image augmentation techniques, including rotation, width and height shift, shear, zoom, and horizontal flip, to diversify the training dataset and improve the models’ generalization capabilities [45]. The fine-tuned models achieved impressive training accuracies, with DenseNet121 leading to the best training accuracy of 99.375%. ResNet50 closely followed with 99.04%, while VGG16 archived a slightly lower with 96.88%. Additionally, the models were evaluated on a separate test dataset containing 2,988 images with an equal distribution of the three classes. The performance metrics of precision, recall, and F_1_-score for each model are summarized below in terms of true positive (TP), true negative (TN), false positive (FP), and false negative (FN). The F_1_-score is calculated as:


$${\text{F}}_{1-score}=\frac{2\times \text{ Recall }\times \text{ Precision }}{\text{ Recall }+\text{ Precision }},$$ (8)

$$\text{ Precision }=\frac{\text{ TP }}{\text{ TP }+\text{ FP }},$$ (9)

$$\text{ Recall }=\frac{\text{ TP }}{\text{ TP }+\text{ FN }}.$$ (10)

**Fig 12:**
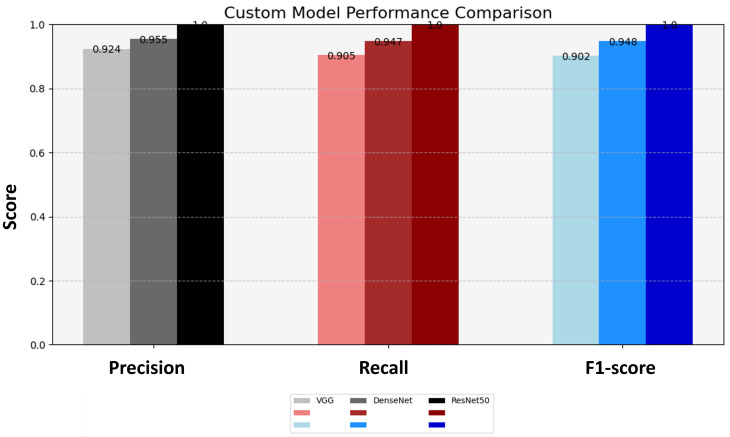
The classification performance metrics. Performance metrics of precision, recall, and F_1_-score for DenseNet121, VGG16 and ResNet50 transfer learning models are compared.

After applying regularization techniques and fine-tuning hyperparameters, DenseNet improved the test accuracy of 99%, with F_1_-score of 0.99 as shown in Fig 12. This shows us a significant reduction between training and test performance, as compared to the initial results (training accuracy 99.37%, F_1_-score 0.9021), indicating enhanced generalization ability.

We conducted statistical analysis using 95% confidence intervals (CI) for the F_1_-score. The results are summarized in Table 3 which showed ResNet50 with F_1_-score of 1.0000 with a very narrow CI (0.995–1.000). DenseNet performed strongly having CI (0.985–0.996) with F_1_-score, while VGG16 obtained a lower score of 0.9476 with CI (0.930–0.962). The non-overlapping intervals between VGG16 and the other models indicate that the observed differences are statistically significant, supporting the claim that ResNet and DenseNet outperform VGG16 in the LifeCLEF-2015 dataset.

**Table 3:** The performance metrics of precision, recall, and F_1_-score for each deep learning model on testing dataset.

Transfer Learning Model	Precision	Recall	F_1_-score	F_1_-score (95% CI)
**VGG16**	0.9549	0.9474	0.9476	(0.930 - 0.962)
**ResNet50**	1.0	1.0	1.0	(0.995 - 1.0)
**DenseNet121**	0.9905	0.991	0.991	(0.985 - 0.996)

In summary, these results demonstrate the effectiveness of fine-tuned models in accurately classifying the underwater images and highlight the superior performance of ResNet50 in this image classification task

## 6. Numerical experimental results

In this comprehensive exploration, our research delved into several critical aspects of underwater video analysis, yielding significant insights and advancements. Below, we provide a detailed account of our experimental findings across various stages of this comparative study on video analysis.

### 6.1. Underwater video exploration

Our meticulous examination of the underwater video dataset unveiled crucial details about its composition and structure. With metadata analysis revealing a vast repository comprising of 9,940 frames, each frame having dimensions of 1920×1012 pixels and a rate of 59.94 frames per second (fps), we gained invaluable insights into the temporal and spatial dimensions of the underwater environment captured in the video. Furthermore, the visual representation of plotting a subset of successive frames provided a compelling visual narrative, offering further context, and setting the stage for subsequent big data processing endeavors.

### 6.2. Image enhancement using Gray World (GW) algorithm

Drawing upon the GW algorithm, we aim to improve the visual fidelity of underwater images, tackling prevalent challenges such as color distortion and inadequate contrast. Through a meticulous application of mathematical adjustments rooted in the L*a*b* color space, our implementation of the GW algorithm yielded significantly improved outcomes. Notably, the color channels were harmoniously balanced, leading to a perceptible enhancement in color equilibrium and contrast within the processed images. Such enhancements are pivotal for discerning finer details and nuances within underwater scenes, consequently enriching the subsequent analysis and interpretation.

### 6.3. Comparison between histogram equalization and CLAHE algorithm

An important novelty of our study is a thorough comparative evaluation between the traditional HE method and the sophisticated CLAHE algorithm. This comparative analysis aimed to discern the efficacy of these techniques in mitigating the inherent challenges prevalent in underwater image processing. Our findings emphasizes the superiority of CLAHE over its conventional counterpart, as evidenced by its ability to preserve intricate image details while reducing undesirable artifacts due to the application of these algorithm.

### 6.4. Edge detection using canny edge detector

Implementing edge detection principles through the Canny edge detector provided a significant milestone in our research, helping us to unveil the intricate contours and boundaries of underwater imagery. Using Gaussian blur and gradient calculations, the Canny edge detector exhibited remarkable insight in identifying the salient features, providing invaluable insights for subsequent analysis. We performed an ablation study by evaluating the performance of the model with and without edge features as shown in Table 4. These results improve classification; ResNet50 achieved a perfect score with Canny edges 1.0 compared to without 0.984, while DenseNet121 improved from 0.975 to 0.991. VGG-16 gained an increase of 0.017. The delineation of edges facilitates object detection and segmentation and increases the interpretability and comprehensibility of underwater scenes, thus fostering a deeper understanding of marine ecosystems.

**Table 4:** Ablation study on the effect of Canny edge detection on classification performance.

Model	With Canny (F_1_-score)	Without Canny (F_1_-score)	Improvement
**VGG16**	0.9476	0.931	+0.017
**ResNet50**	1.000	0.984	+0.016
**DenseNet121**	0.991	0.975	+0.016

### 6.5. Transfer learning-based image classification

**Fig 13:**
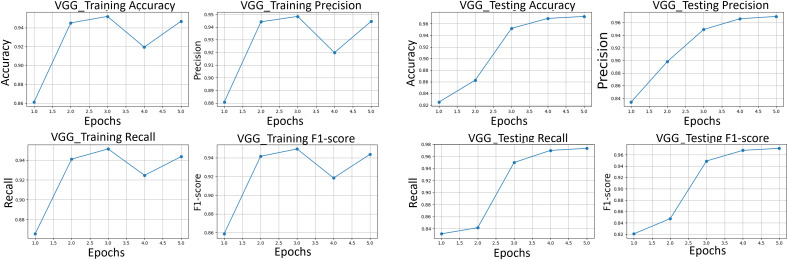
VGG. (*left)* VGG training performance, *(right)* VGG testing performance of accuracy, precision, recall, and F_1_-score.

**Fig 14:**
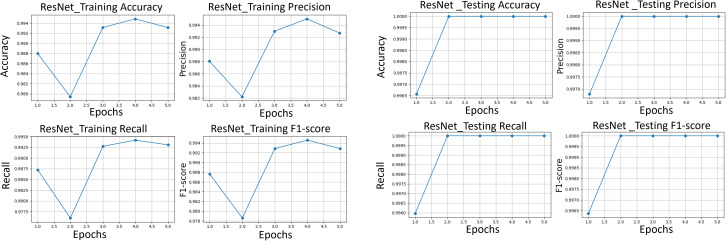
ResNet training performance. The performance of accuracy, precision, recall, and F_1_-score (*left)* and testing performance *(right)*.

**Fig 15:**
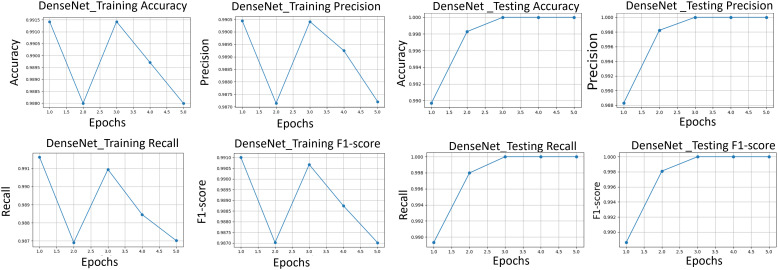
DenseNet training performance. of accuracy, precision, recall, and F_1_-score (*left)* and testing performance *(right)*.

**Fig 16:**
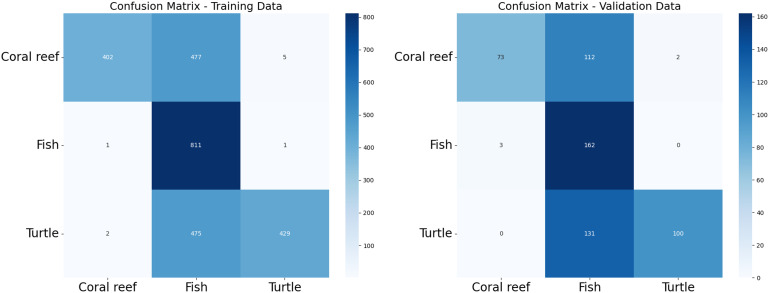
VGG confusion matrix. *(left)* VGG confusion matrix using training data, *(right)* VGG confusion matrix using testing data.

**Fig 17:**
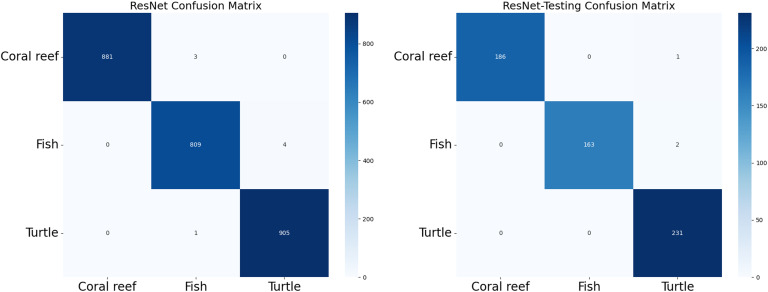
ResNet confusion matrix. *(left)* ResNet confusion matrix using training data, *(right)* ResNet confusion matrix using testing data.

**Fig 18:**
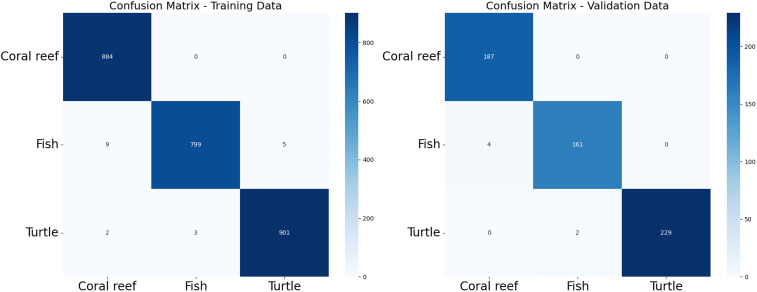
DensNet confusion matrix. *(left)* DenseNet confusion matrix using training data, *(right)* DenseNet confusion matrix using testing data.

**Fig 19:**
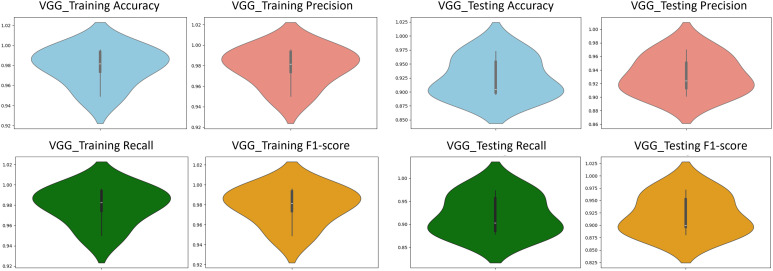
VGG violin plots. The plots showing accuracy, precision, recall, and F_1_-score using training data *(left)* and testing data *(right)*.

**Fig 20:**
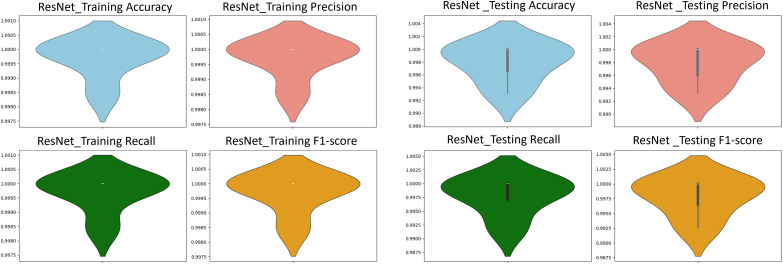
ResNet violin plots. The plots showing accuracy, precision, recall, and F_1_-score using training data *(left)* and testing data *(right)*.

**Fig 21:**
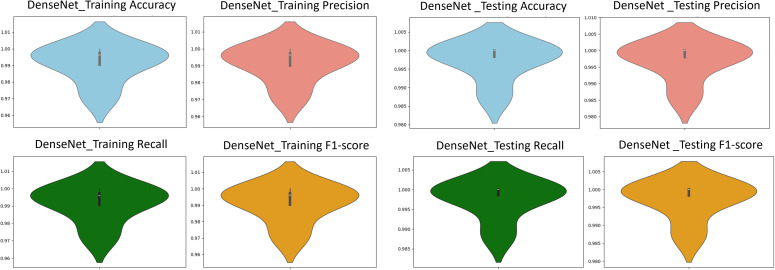
DenseNet violin plots. The plots showing accuracy, precision, recall, and F_1_-score using training data *(left)* and testing data *(right)*.

To harness the transformative potential of transfer learning, we explored three pre-trained CNN architectures: VGG16, ResNet50, and DenseNet121. We use a multifaceted approach to discern marine biodiversity by fine-tuning these models for image classification tasks of fish, coral reef, and sea turtle categories. With a meticulously curated dataset comprising over 30,000 images, our models focused on training and validation, paving the way for valuable insights into the classification process. The robust performance exhibited by DenseNet121, culminating in a enthusiasm training accuracy of 99.375%, underscored the efficacy of transfer learning in supporting image classification tasks within the marine biodiversity assessment domain. The commendable performance of ResNet50 and VGG16 further corroborated the transformative potential of transfer learning to allow accurate and discerning classification of aquatic organisms. As shown in Figs 13-15, the training and testing performance curves are illustrated by the convergence behavior in VGG16, ResNet50 and DenseNet121. Confusion matrices were used to emphasize the class-wise prediction and misclasiffication as shown in Figs 16-18. In addition, violin plots shown in Figs 19-21 highlighted the distribution of the performance for each class, enabling a more nuanced comparison of the robustness of the model. Table 5 display comparisons cross various fish species and classification models performance in different benchmarks.

**Table 5:** Comparison between results of underwater image/video classification models.

Reference | Year	Dataset	Machine Learning Model	Metric	Result
**Fish**				
**[49] 2025**	SEAMAPD21	YOLOv8, Transformer	mAP@0.5/0.5:0.95	87.9/61.2
**[13] 2024**	Freshwater fish dataset	DeformableFishNet	mAP	96.3
**[42] 2023**	Mediterranean fish dataset	YOLOv5m	mAP@0.5	84.0
**Coral reef**				
**[50] 2024**	Coral image dataset	Swin-B, EfficientNet-B7	Macro-F1	81.7
**[51] 2024**	Indonesian coral reef	YOLOv8	Accuracy	97.8
**Turtle**				
**[52] 2024**	iNaturalist	YOLOv5x-seg	mAP@0.5/0.5:0.95	91.8/83.1
**[53] 2024**	SeaTurtleID2022	ResNet50, Swin-B	mPA	86.8
**Fish, Coral reef, Turtle**				
**[45] 2024**	Video 9,940 images	YOLOv8	Accuracy	99.0
**[7] 2025**	LifeCLEF-2015	DenseNet121, ResNet-LSTM, Vision Transformer	Accuracy	85.1, 88.3, 85.9
**Current work 2026**	Video (9,940)	VGG16, ResNet50, DenseNet121	Accuracy	96.9, 99.0, 99.4
**Current work 2026**	LifeCLEF-2015	VGG16, ResNet50, DenseNet121	Accuracy	94.8, 99.0, 99.6

## 7 Image enhancement using GAN

**Fig 22:**
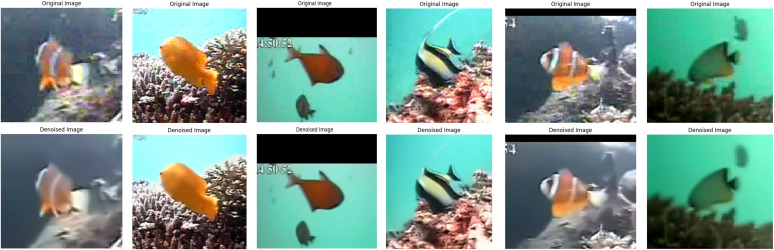
Stages of image enhancement. First row are original images, and second row are denoised images. This method improves edge accuracy by removing noise and keeping important details.

The focus of this section is to improve the quality of noisy images using state-of-the-art image processing methods such as GAN models, specifically using SRGAN and ESRGAN. Through these techniques, the video processing pipeline is improved by underwater image denoising, generating images with super resolution, and post-processing to produce high-quality visuals [11,40]. To illustrate the performance of the image enhancement techniques using GAN, the LifeCELF-2015 dataset has been used as a benchmark [44]. This dataset contains a range of underwater images with low quality of lighting and contrast, making it a good choice for evaluating SRGAN and ESRGAN. Preprocessing is a step that involves cleaning up noisy images. The process takes place before the resolution of these images is improved using GAN. Fast non-local means denoising is a technique that is used to remove noise from images by considering the colors of a neighboring pixel and then deciding the best color for each pixel in the noisy image, as shown in Fig 22. This technique removes noise while maintaining important details such as the sharpness of the edges [54].

SRGAN and ESRGAN are advanced deep learning methods that prove the quality of blurry images and enhance image resolution [11,12]. According to [55], these have two main parts: firstly, the generator (G) that improves the low-resolution (LR) images by turning them into high-resolution (HR) images. Secondly, a discriminator (D) that tells the difference between images that are originally high quality and those enhanced using the generator. The generator utilizes perceptual loss to create images that look similar to the ground truth. The content loss for SRGAN is described as follows:


$${ℒ}_{\text{content }}=\frac{1}{WHC}\sum_{i,j}{\left\|\phi_{i,j}\left(I^{\text{HR}}\right)-\phi_{i,j}\left(I^{\text{SR}}\right)\right\|}^{2}.$$ (11)

Here, W H C refers to the dimensions of the feature map, which represent the *W* (width), *H* (hight), and *C* (number of channels) of the feature map. *I*HR and *I*SR are the ground truths and generated images respectively, and $\phi_{i,j}$ is the feature map from the *i*^*th*^ layer. The SRGAN includes an adversarial loss to motivate the generator to create realistic images [55]:


$${ℒ}_{\text{adversarial }}=-{𝔼}_{I^{\text{SR}}}\left[\log\left(D\left(I^{\text{SR}}\right)\right)\right].$$ (12)

The total generator loss is weighted sum of the content loss and adversarial loss:


$${ℒ}_{\text{generator }}={ℒ}_{\text{content }}+\lambda {ℒ}_{\text{adversarial }}.$$ (13)

In the construction of SRGAN, ESRGAN provides an improvement to enhance the quality of the images. These include the application of Residual-in-Residual Dense Blocks (RRDB) in the generator to improve the capacity of the network to learn high-frequency details and to prevent degradation during training. ESRGAN uses a relativistic GAN loss to yield more realistic images. The adversarial loss in ESRGAN and the total generator loss are described as:


$$\begin{array}{c} {ℒ}_{\text{relativistic }}=-{𝔼}_{I^{\text{H R}}}\left[\log\left(D\left(I^{\text{H R}}-D\left(I^{\text{S R}}\right)\right)\right)\right]\\ -{𝔼}_{I^{\text{S R}}}\left[\log\left(1-D\left(I^{\text{S R}}-D\left(I^{\text{H R}}\right)\right)\right)\right], \end{array}$$ (14)

$${ℒ}_{\text{generator }}={ℒ}_{\text{content }}+\lambda {ℒ}_{\text{relativistic }}.$$ (15)

**Fig 23:**
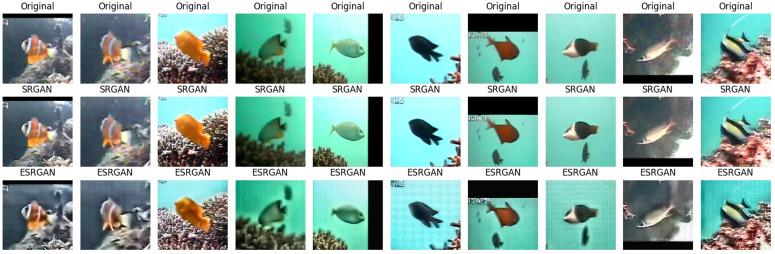
Results for 10 random samples of LifeCLEF-2015 dataset. First row shows original images, second row shows enhancement images using SRGAN techniques, and third row shows enhanced images using the ESRGAN technique.

The SRGAN produced realistic images and detail enhancement more than ESRGAN as shown in Fig 23. We aim to evaluate model performance using objective metrics such as the Peak Signal-to-Noise Ratio (PSNR), the Structural Similarity Index (SSIM), and Mean Squared Error (MSE) [56]. These measures will allow us to provide a more rigorous comparison between the ESRGAN and SRGAN methods, and to validate improvements in perceptual quality with numerical evidence of image enhancement. The evaluation of both GAN models indicates that SRGAN achieves lower MSE than ESRGAN, and higher PSNR. These results show that SRGAN generates improved underwater images that are nearly to HR ground truth, whereas the ESRGAN achieves higher MSE, lower PSNR and lower SSIM as shown in Table 6. These results suggest that ESRGAN is less effective than SRGAN in underwater image enhancement.

**Table 6:** Comparison of SRGAN and ESRGAN Enhancement Performance.

Metric	SRGAN	ESRGAN
**MSE**	0.1795	0.2003
**PSNR (dB)**	7.5830	7.0556
**SSIM**	0.5720	0.3477

## 8. Conclusion and future works

This paper shows valuable insights and advancements in underwater video analysis through transfer learning models. Using systematic exploration of various techniques and methodologies, we have achieved significant progress in enhancing underwater image quality and facilitating accurate classification of marine species. Key findings from our study include the effectiveness of the Gray World algorithm in addressing color distortion and contrast issues, the superiority of CLAHE over traditional HE for image enhancement, and the successful application of the Canny edge detector for feature extraction in underwater image processing. Moreover, the transfer learning-based approach, leveraging pre-trained CNN models such as VGG16, ResNet50, and DenseNet121, have demonstrated remarkable performance in image classification tasks, while the DenseNet121 emerged as the most accurate underwater image classifier. The significance of our work lies in its potential to advance marine science and environmental monitoring efforts through improved underwater video analysis techniques. By enhancing the visual quality of underwater images and enabling precise identification of marine species, our research contributes to a deeper understanding of aquatic ecosystems and supports conservation initiatives. SRGAN is an excellent GAN technique that was used to improve the quality of blurry and noisy images as it sharpened the details without removing any of the essential information from the images. We used open dataset such as LifeCLEF-2015 [57], for our analysis. This dataset consists of images extracted from different underwater videos with limited species, behaviors, and water conditions. Therefore, this dataset is a challenging benchmark for enhancing underwater images. Moreover, the GW algorithm supposed to balance the color distribution which was a failure with lighting reflection and shadows. The GW is limited and struggles to correct and shift the colors, often leading to unnatural results. Also, the GW struggles with low lighting conditions where the intensity of images in dark shadows is poor, resulting in poor color correction. In the future research may focus on addressing these identified limitation by improving both underwater image enhancement and classification performance. First, for enhancement, training deep model such as GAN should use underwater datasets that handle underwater color distortion and low contrast conditions to improve SSIM and reduce the distortions. In addition, the training dataset should contain greater environmental realism, underwater video with motion, depth and multiple species for increasing model robustness. Second, for classification, future researches may address class imbalance and rare species recognition by training data and learning the strategies.

In summary, our work represents a significant step forward in underwater video analysis, offering valuable contributions to scientific knowledge and practical applications in marine biology, ecology, and environmental conservation. By continuing collaboration between machine learning and ecology, we can unlock new opportunities to understand and preserve the diverse ecosystems of our oceans for future generations.
